# Perspectives of TRPV1 Function on the Neurogenesis and Neural Plasticity

**DOI:** 10.1155/2016/1568145

**Published:** 2016-01-05

**Authors:** R. Ramírez-Barrantes, C. Cordova, H. Poblete, P. Muñoz, I. Marchant, F. Wianny, P. Olivero

**Affiliations:** ^1^Escuela de Medicina, Universidad de Valparaíso, Hontaneda 2664, 2341386 Valparaíso, Chile; ^2^Institute of Computational Comparative Medicine, Nanotechnology Innovation Center of Kansas State, Department of Anatomy and Physiology, Kansas State University, Manhattan, KS 66506-5802, USA; ^3^INSERM, U846, Stem Cell and Brain Research Institute, 18 Avenue Doyen Lépine, 69500 Bron, France; ^4^Université de Lyon, 69003 Lyon, France

## Abstract

The development of new strategies to renew and repair neuronal networks using neural plasticity induced by stem cell graft could enable new therapies to cure diseases that were considered lethal until now. In adequate microenvironment a neuronal progenitor must receive molecular signal of a specific cellular context to determine fate, differentiation, and location. TRPV1, a nonselective calcium channel, is expressed in neurogenic regions of the brain like the subgranular zone of the hippocampal dentate gyrus and the telencephalic subventricular zone, being valuable for neural differentiation and neural plasticity. Current data show that TRPV1 is involved in several neuronal functions as cytoskeleton dynamics, cell migration, survival, and regeneration of injured neurons, incorporating several stimuli in neurogenesis and network integration. The function of TRPV1 in the brain is under intensive investigation, due to multiple places where it has been detected and its sensitivity for different chemical and physical agonists, and a new role of TRPV1 in brain function is now emerging as a molecular tool for survival and control of neural stem cells.

## 1. Introduction

*Repairing and Renewing the Brain from the Inside*. Brain development implicates cell migration, differentiation, and plasticity to configure an efficient neuronal network. While neurons can live long periods of time, a large number of neurons die during developmental and pathophysiological processes in a lifetime. The loss of neurons in adulthood can lead to nervous system disorders such as neurodegenerative diseases, which involve cognitive and motor alterations causing severe disability and generally death. While pharmacological treatment for this kind of diseases may attenuate symptoms and disease progression at initial stages, pharmacological efficacy gradually decreases over time [1]. New experimental approaches must be developed to design efficacious therapies for repairing and renewing the neuronal network to restore lost functions in order to expand possibilities of cures for brain diseases.

Neural stem cells (NSCs) can generate different types of neurons. In order to generate sensory neurons, motor neurons, or interneurons, NSCs in adequate microenvironment must receive cellular context-specific molecular signals to determine fate and location. These newly formed neurons establish new circuits and modify existing pathways connecting neuron to neuron. In this context, NSC graft appears to be a promising strategy to recover lost functions due to neurons death in the brain. Unfortunately, neural grafts have not been as successful as expected, due to poor survival of grafted cells and the inability of surviving cells to connect with central network [2, 3]. The control of stem cells differentiation into specific types of neurons as well as their survival and connectivity may enable the establishment of a renewal supply to replace dead or damaged neurons. Recently, the controlled expression of the nonselective cationic channel TRPV1, which is widely expressed in peripheral and central nervous system [4–9], has proven effective on the control of many functions in brain neurons [10–12]. The regulated activity of TRPV1 promotes migration [13], axon growth [14], cell-to-cell extension [15], and release of neurotransmitters (GABA, dopamine, and glutamate) [6, 16], and more interestingly a novel function has been reported which is the ability to control brain neurogenesis [10, 17, 18]. In addition, the regulated activation of the channel is also involved in cell resistance against local oxidative environment in brain regions and other tissues [11, 19–21].

In this review we explore the contribution of TRPV1 channel function in NSC fate, discussing possible roles of the channel in neurogenesis and network integration, and eventually we propose the use of TRPV1 control as a new clinically promising strategy to improve the plasticity of newborn neural network derived from grafted neural precursors in the damaged brain.

## 2. Polymodal TRPV1: An Environmental Signal Integrator

**Figure 1:**
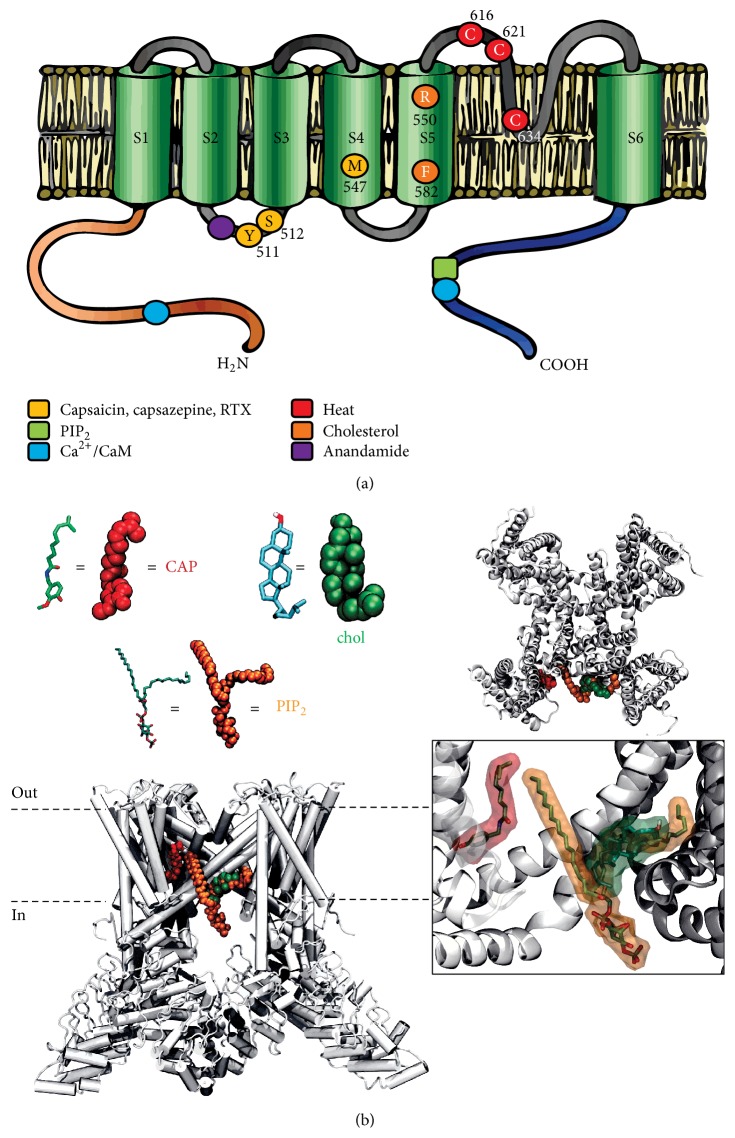
Diagram of regions involved in TRPV1 function. (a) The primary structure involves six transmembrane segments (S1–S6) with a pore domain between the fifth (S5) and sixth (S6) segment, and both C and N termini are located intracellularly. The functional TRPV1 receptor is believed to form a homotetramer. Amino acid residues involved in the binding of chemical and physical activation/modulation of TRPV1 activity are indicated in a color scheme. Vanilloid compounds, as the activators capsaicin and resiniferatoxin, as well as inhibitor capsazepine share the same binding site, while cholesterol-binding site is composed of a promiscuous hydrophobic pocket in S5. (b) Model for hydrophobic pocket in S5 linker with the binding of lipidic molecules such as cholesterol (chol), PIP_2_, and capsaicin (CAP) generated by molecular dynamics. In this binding conformation, all the molecules occupy a groove formed between S5 and C-terminal of the subunit.

Transient receptor potential ion channel (TRP) family consists of a number of nonselective cationic channels capable of integrating environmental physicochemical signals and coupling their activity to downstream amplification of cellular signal through cation permeation and membrane depolarization [4, 9, 22–24]. In cellular context, the activity of TRPs is modulated by several molecular mechanisms such as phosphorylation, reactive oxygen species (ROS), membrane lipid composition, intracellular calcium, and ATP levels [8, 24–26]. Among the six members (TRPV1-6) of TRPV (vanilloid) subfamily, TRPV1 was the first identified and best characterized [8, 27]. TRPV1 is a homotetrameric nonselective cation channel (*P*_Ca_/*P*_Na_ = 9.6) with the same characteristics as other TRPs [28]. It is activated by several physical stimuli such as temperature, voltage, protons, osmolarity, pH [4, 23, 27], chemical ligands such as PIP_2_ or endocannabinoids like anandamide, and exogenous ligands as resiniferatoxin and capsaicin [27, 29, 30] (Figure 1(a)).

New evidence links a specific hydrophobic pocket near the S5 helix that contains amino acids R579, F582, and L585 to the binding of PIP_2_ [31] and cholesterol [32] (Figure 1(b)). The binding of these two molecules to the hydrophobic pocket may either potentiate or inhibit TRPV1 activity. More interestingly, the *α*-3-OH diastereoisomer of cholesterol epicholesterol has no effects on TRPV1-mediated currents, suggesting the existence of a stereospecific binding site [32]. 17*β*-Estradiol increases currents evoked by capsaicin in dorsal root ganglion neurons [33] and capsaicin-induced nociception, whereas these capsaicin effects are reduced by testosterone [34]. Thus, modification of TRPV1 hydrophobic environment may alter its biophysical properties and contribute to functional coupling. Several mechanisms show intrinsic cooperative regulation, suggesting allosteric modulation of these ion channels, although there is no definite evidence on the potential binding site. It is noticeable that, in particular context, TRPV1 could work as ionotropic receptor of cholesterol-derived molecules with opposite actions. This feature allows them to act as signal integrators [31], playing critical roles in excitable and nonexcitable cell functions underlying sensory physiology, proliferation, growth, male fertility, and neuronal plasticity [7, 35–37].

## 3. TRPV1 in the Brain

TRPV1 was first described in peripheral afferent fibers and identified as a detector of harmful signals in primary sensory neurons [27]. The currently known distribution of TRPV1 includes testis, heart, lung, stomach, and brain [8, 36, 38–40]. Particularly, in peripheral nervous system (PNS) TRPV1 is highly expressed in dorsal root ganglia (DRG), trigeminal ganglia, and primary sensory neurons, which are sensory neuronal components of nociceptive A*δ* and C-fibers' circuits [4, 8]. In PNS TRPV1 was primarily studied for its anti-inflammatory and antinociceptive functions [20, 27, 37], but currently a more general function has been attributed to TRPV1; this is an integrator of several noxious stimuli such as low pH (pH < 6.0) or high temperature (>43 degrees Celsius) [41]. In central nervous system (CNS) the expression of TRPV1 is still controversial. Whereas some seminal reports showed very low or no expression of the channel in CNS [27, 42], recent reports have shown (1) that well-recognized endogenous activators such as N-arachidonoyldopamine (NADA) or exogenous activators such as capsaicin (CAP) or even potent TRPV1-specific inhibitors like capsazepine (CPZ) or resiniferatoxin (I-RTX) can modulate the activity of neurons in CNS [11, 36, 43, 44] and (2) direct evidence on the expression of TRPV1 by immunohistochemistry, PCR, autoradiography, and* in situ* hybridization in mammalian brain [5, 39, 45, 46]. The amount of expression of TRPV1 differs importantly between central and peripheral nervous system. In the brain, it is 20- to 30-fold lower than in DRG [27, 47]. The poor TRPV1 expression in CNS has demanded greater precision and refinement of experimental methods in order to increase the reliability of localization of the channel in the brain and its significance. In addition, the existence of TRPV1 alternates which are heterogeneously distributed throughout the nervous system [48] complicates the interpretation of the results from several expression studies. However, a remarkable study using mice with genetically modified TRPV1 reporter protein along with other techniques such as* in situ* hybridization, calcium-imaging, RT-PCR, and slice electrophysiological recordings provided definite evidence on the expression of functional TRPV1 in primary afferent neurons while low levels of expression were found in entorhinal cortex, olfactory bulb, hippocampus, and hypothalamus [43], which are nevertheless active enough to modulate excitability in hypothalamus [43]. More intriguingly, TRPV1 can be transiently expressed during brain development. In some brain regions the expression can suffer postnatal restriction depending on age, physiological, or pathological condition [45], suggesting that TRPV1 functional expression might be modulated by the metabolic cell state.

**Figure 2:**
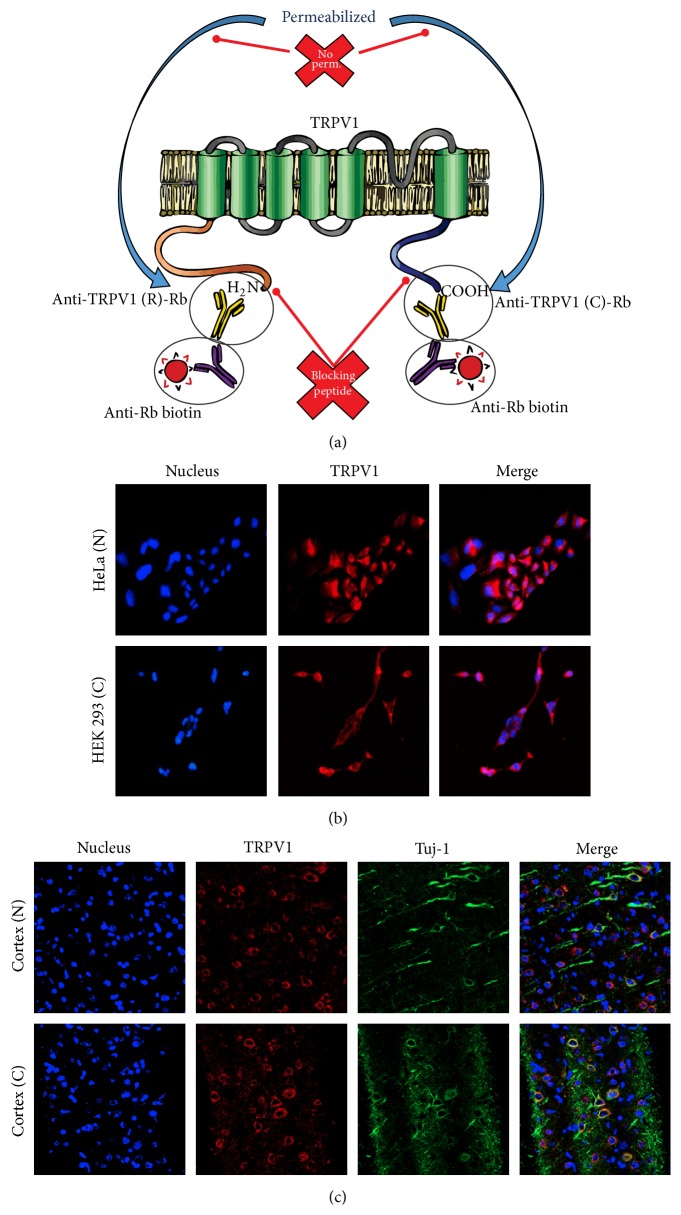
TRPV1 detected by immunofluorescence. (a) Methodology proposed for detection of TRPV1 by immunofluorescence. Using two antibodies against different epitopes of the channel allows corroborating the expression of the channel. In this case, we showed an antibody against the C-terminal and another against the N-terminal. As both antibodies bind to intracellular epitopes, it is advisable to use as internal control of the technique a sample without permeabilization of the plasma membrane, which prevents the entry of the antibody into the cell. One added strategy to improve signal sensitivity was the use of a blocking peptide, in this case, for the C-terminal or N-terminal. The competition of the blocking peptide with the epitope of the channel should diminish the intensity of the signal indicating the specificity of the technique. (b) Detection of TRPV1 in heterologous expression system using antibodies against the N-terminal and C-terminal of TRPV1. (c) Detection of TRPV1 in primate prefrontal cortex, using an antibody against the N-terminal and another against the C-terminal.

The number of reports addressing the functional effect of activation/suppression of TRPV1 channel expressed in several brain regions increases each year. To date, both TRPV1 mRNA and protein have been found mainly in cortical structures and hippocampal pyramidal neurons in areas CA1, CA3, and dentate gyrus but have also been found in the locus coeruleus, cerebellum, thalamic and hypothalamic nuclei, periaqueductal grey, and limbic structures including the caudate putamen, the central amygdala, and the substantia nigra pars compacta [5, 45, 49]. With regard to the cell type where TRPV1 is expressed, it has been reported in different lineages, most commonly neurons. For instance, in hippocampal dentate gyrus many pyramidal neurons throughout the CA1–CA3 areas express TRPV1 receptor on cell bodies. In thalamus, TRPV1 expression has been found in neuronal cytoplasmic and axonal staining; in cerebellum TRPV1 channels surround several Purkinje cell bodies, especially on basal areas corresponding to the initial axonal segment; in cortex the expression also surrounds the nucleus; and in substantia nigra double labelling immunofluorescence shows a complete overlap between TRPV1 and tyrosine hydroxylase, confirming the presence in dopaminergic neurons [5, 46, 50]. We assayed our experimental strategy to identify the expression of TRPV1 by immunofluorescence in heterologous system and in neurons of primate prefrontal cortex, confirming the expression of TRPV1 in neurons and glia in mammalian brain (Figure 2).

## 4. TRPV1 Expression in Neural Progenitors

**Figure 3:**
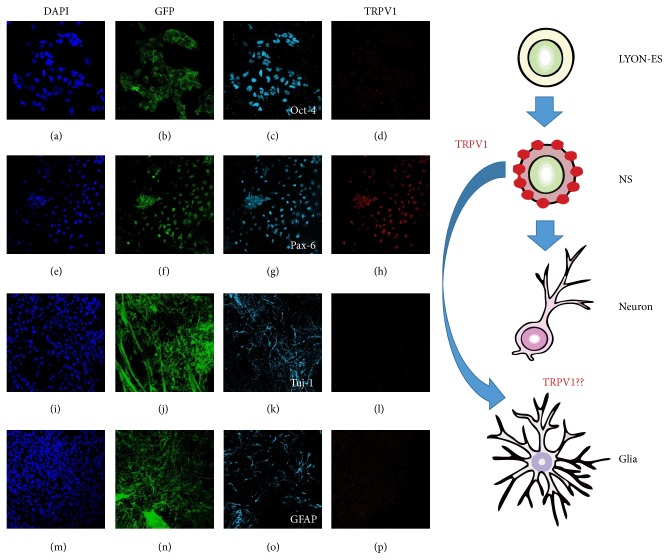
Expression of TRPV1 in neuronal differentiation process derived from LYON-ES. Determination of expression of TRPV1 by immunofluorescence staining for undifferentiated monkey ESCs stably expressing Tau-GFP (TAU-GFP LYON-ES1 line, a–d), NSCs (NS, e–h), neurons (i–l), and glial cells (m–p) derived from TAU-GFP-LYON-ES1 cells. At each stage, we performed immunofluorescence with antibodies against Oct4 (c) to identify LYON-ESCs, Pax6 for NSCs (g), *β*-III-tubulin for neurons (k), glial fibrillary acid protein (GFAP) for glial cells (o), and TRPV1 (d, h, l, p). Each experiment was accompanied by nuclear staining with DAPI (a, e, i, m), all the cells having a GFP fused to the microtubule-associated protein tau (b, f, j, n). The magnification of images was 20x for LYON-ESCs and neurons, and 40x for NSCs and glia cells.

Recent publications add a novel cell lineage to the vast list of cell types that express this channel in the brain. TRPV1 is expressed in neurogenic brain regions, in particular, in the hippocampal dentate gyrus subgranular zone (SGZ) as well as the subventricular zone in telencephalon (SVZ). In adult rat, TRPV1 is colocalized with nestin, a marker of NSCs. Since postnatal neurogenesis occurs up to day 21 and declines afterwards in mice, the expression of TRPV1 was measured in postnatal days 7, 14, 21, and 39, being positive at the time points that corresponded to the time course of postnatal neurogenesis p7, p14, and p21. More interestingly, TRPV1 was no longer detected from p39, when postnatal neurogenesis had declined [18]. Additionally to the expression of TRPV1 detected in early neural precursors, stimulating neurogenesis by exercise paradigm upregulates TRPV1 expression above baseline in the adult hippocampus [18]. In the same line, we induced* in vitro* differentiation of monkey embryonic stem (ES) cells to neural precursor and explored the expression of TRPV1 at different stages of differentiation. We used the LYON-ES1 cell line that stably expresses Tau-GFP, isolated at SBRI (Stem Cell and Brain Research Institute, INSERM, France). The primate pluripotent markers-expressing LYON-ES1 cells [51] are indefinitely self-renewable and have the capability of multilineage differentiation [51, 52]. We examined the expression of TRPV1 in LYON-ES1 cells, NSCs, glial cells, and neurons derived from LYON-ES1 cells. We used Pax6 as a marker for NSCs, *β*-III-tubulin, and glial fibrillary acidic protein (GFAP) as markers for neurons and glial cells [51, 52], respectively. We found that TRPV1 was expressed in NSCs (Figure 3) with cytoplasmic signal accompanied by a nuclear mark (Figure 3), as described previously [46, 53, 54]. In contrast, we did not detect TRPV1 in pluripotent stem cells, neurons, or glial cells.

The expression of TRPV1 has also been characterized in specific regions of the brain as previously mentioned [16, 36, 39], with particular microenvironment or extracellular pathways engaged in neurogenesis. The expression of TRPV1 in NSCs was evident in all experiments supporting the* in vivo* results previously reported [18].

Up until now, the TRPV1 expression pattern in neural precursors and its role in neurogenesis have been poorly studied and a new field on TRPV1 research is open with interesting implications in tissue regeneration.

## 5. TRPV1 Functions in the Brain

The function of TRPV1 in the brain has been subjected to exhaustive investigation, because of the multiple places where it has been detected, its sensitivity to different chemical and physical agonists, and its versatility as calcium channel. The intracellular calcium concentration modulated by TRPV1 is capable of triggering various processes such as excitability, proliferation, synaptic plasticity, resistance to oxidative stress, and cell death, depending on the concentration, timing, and transience of the signal [49, 55].

The most studied aspect of TRPV1 function relates to its activity on synaptic plasticity and excitability in the brain. The control of TRPV1 activity has proven effective to modulate the excitability in neurons [56–58]. In particular, peripheral nerve endings increase glutamate release following the activation of TRPV1 by heat [27]. Capsaicin, NADA, and endocannabinoids increase release of neurotransmitter in the central nervous system, the basal ganglia, hypothalamus [6, 12, 59], and cranial visceral afferent terminals in caudal solitary tract nucleus (NTS), in brainstem [60]. TRPV1 is involved in hippocampal long-term potentiation (LTP) [61] and depression (LTD) mediated, respectively, by vanilloids and endocannabinoids like anandamide [36, 59]. In addition, it could induce release of GABA in dentate gyrus depressing excitatory synaptic transmission [62]. Lastly, in the neighboring ventral tegmental area, capsaicin also increased the firing rate of dopamine neurons [16] as it did in excitatory synapses in the substantia nigra. Facilitated spontaneous excitatory postsynaptic current frequency by capsaicin and NADA without affecting amplitude suggested a presynaptic mechanism [63, 64].

TRPV1 has recently been demonstrated to have an important role in the regulation of cortical excitability by modulation of synaptic transmission in the human brain [58]. It would be interesting to understand how several physical and chemical activators or modulators interact to enhance or inhibit TRPV1 activity, because an allosteric coupling has been demonstrated with distinct agonist, increasing the effect of the channel activation and stimulating neuroplasticity [29, 31, 32].

## 6. TRPV1 and Neurogenesis

Spontaneous calcium oscillations play an important role in nervous system development, neural induction, axon guidance, growth cone morphology, migration, and proliferation [15, 65–67]. These oscillations are a combination of extracellular influx mediated by ionic channels and release of intracellular store from endoplasmic reticulum and/or mitochondria. TRP channels like TRPC and TRPV family have been related to control of neuronal differentiation and activity in CNS. TRPC channels mediate cortical neural precursor proliferation induced by bFGF [68] and TRPV1 differentiation triggered by retinoic acid [10] and rimonabant [17]. These two families also modulate the excitability in well-differentiated neurons and their precursors.

The first signs that linked TRPV1 with neurogenesis were indirect; in transiently transfected F11 cells and embryonic DRG neurons that endogenously express the channel, it was localized in neurites and growth cones where it regulates motility [15]. Dynamic processes such as growth cone motility and the direction of neurites involve calcium signals via cell surface receptors. The guidance of developing axons requires an active growth cone, and a localized calcium signal in the growth cone is sufficient for both attraction and repulsion [69]. TRPV1 located in the growth cone has been involved in the formation of filopodia in neurons [15]. In addition, TRPV1 contributes to cytoskeleton reorganization [70], cell migration [13], and regeneration of injured neurons [53].

These early discoveries indicate a key role of TRPV1 during neuronal differentiation. More specifically, TRPV1-expressing SHSY5Y neuroblastomas induced to differentiate by retinoic acid showed upregulation of total and cell surface TRPV1 protein expression. Specifically, these upregulated channels were localized in cell bodies and the new neurites. Besides, retinoic acid increased both the intracellular free calcium concentration and the relative calcium influx induced by capsaicin [10]. Moreover, rimonabant, an antagonist of cannabinoid receptor 1 (CB1), was evaluated as inducer of neurogenesis in dentate gyrus and subventricular zone (SVZ), expecting that the inhibition of CB1 triggered the generation of neurons [17]. Neurogenesis was increased in both wild-type and knockout mice for CB1, but the neurogenesis-promoting effect of rimonabant disappeared in TRPV1 knockout mice [17]. Until now the mechanisms remain unexplored, although the interaction of rimonabant and TRPV1 has been associated with other processes as neural cell survival in a global cerebral ischemia model [21]. The hypothesis is that the quick activation of TRPV1 followed by desensitization could induce transient increase of calcium signal activating survival pathways and others associated with neurogenesis such as ERK pathway [17]. At least in the case of neuroprotection, the effect over TRPV1 appears to be direct since it is abolished by the application of CPZ [21].

These findings added evidence on the TRPV1 involvement in neurogenesis and the interaction between vanilloids and the endocannabinoid system during the generation of new neurons. However, in dentate gyrus and the subventricular zone, loss of TRPV1 expression promotes proliferation of neural precursors. The TRPV1 knockout mice exhibited substantial rise in postnatally proliferating cells in both stem cell niches, but lesser differentiation to neurons or glia [18]. The primary neural precursors originated from newborn TRPV1 knockout mice expressed stem cell genes like nestin or Sox2 and no differentiation markers for astrocytes (GFAP) or neurons (*β*-III-tubulin). Thus, the loss of TRPV1 in neural precursors disturbs differentiation and the growth potential. These data confirm the role of TRPV1 in orchestrating proliferation/differentiation of neural precursors, which has already been reported in other cell types.

Finally, another interesting and not well-understood function of TRPV1 is the control of cell death. Micromolar concentrations of capsaicin and acid solution (pH 5.5) induce a cytosolic calcium increase, mitochondrial membrane depolarization, ROS production, and cell death via TRPV1 activity [71]. In rat cortical neurons TRPV1 activation by capsaicin induces apoptotic cell death through L-Type Ca^2+^ channels, provoking Ca^2+^ influx, ERK phosphorylation, ROS production, and caspase-3 activation [72]. However, similar results have been reported for capsaicin without TRPV1 participation [73, 74] suggesting both dependent and independent effects of this vanilloid. TRPV1 knockout (KO) mice present a testis, brain, and heart phenotype much more susceptible to cell death by oxidative stress stimuli compared to wild-type mice [40, 75]. Pretreatment with capsaicin can prevent cell death induced by ischemia/reperfusion in lung in rabbits with concomitant diminishing of lipid peroxidation [76]. Besides, in hippocampus subjected to 10 min ischemia, CA1 neurons pretreated with capsaicin were less susceptible to cell death and the effect was inhibited with capsazepine antagonist of TRPV1. The use of rimonabant, the same compound that induces neurogenesis by TRPV1, as a postischemic treatment facilitated neuroprotection independent of CB1 receptor and inhibited by capsazepine. The same effect was measured in a model of temporary global cerebral ischemia by pretreatment with capsaicin in Mongolian gerbils [20]. The mechanism suggested involves a moderate increase in Ca^2+^ influx via TRPV1. This transitory influx may induce tolerance to subsequent calcium overload, preconditioning the response and inducing neuroprotection. However, capsaicin administrated 5 minutes after recirculation had no effect [20]. A possible explanation is that capsaicin and other pharmacological agonists of TRPV1 induce activation of the channel followed by acute desensitization. In the case of vanilloids, this could occur after the first 20 seconds following the addition of vanilloid compounds [77]. The molecular mechanism includes several pathways related with intracellular Ca^2+^ concentration. One of them is dependent on the balance between phosphorylation and dephosphorylation of TRPV1 triggered by Ca^2+^-calmodulin pathway. Moreover, the dephosphorylation of the amino acid Ser502 and Thr704 by CaMKII has been associated with desensitization of the channel [77]. On the other hand, it has been demonstrated that calcium may induce TRPV1-caveolar endocytosis and lysosomal degradation [78, 79]. Independently of the mechanisms, the controlled activity of this polymodal receptor (activation-desensitization) seems to be critical for the cellular homeostasis in oxidative environment, acting as a modulator of cell viability. The precise cellular mechanism underlying TRPV1 activation-modulating cell homeostasis and viability remains unclear. Current information on cell death relates the activation of TRPV1 with abnormal function of the mitochondria. Mitochondrial dysfunction is frequently observed in cell death induced by high doses of capsaicin through TRPV1 activation. A significant portion of calcium entering the cytoplasm after the activation of TRPV1 is accumulated by mitochondria. Uptake of calcium by DRG neurons rises up to 20-fold compared to controls in the presence of 1 *μ*M capsaicin, without observable cytotoxic effect; however, pretreatment with the mitochondrial uncoupler almost stopped capsaicin-dependent accumulation of calcium [80, 81]. Furthermore, TRPV1 expression diminishes the damage produced by high salt-diet in mouse heart compared to knockout maintaining the mitochondrial function [82]. Particularly, the controlled activation of TRPV1 induces an increase in expression of sirtuin 3, a protein that regulates the activity of Complex I, ATP production, and increases ROS clearance through deacetylation of Mn-SOD [83]. It seems that capsaicin activation of TRPV1 can prevent cardiac mitochondria dysfunction caused by high salt intake [82]. Apparently, the activity of TRPV1 is coupled to mitochondrial function, regulating the calcium buffering and the clearance of mitochondrial ROS [84]. The deregulated activation of TRPV1 by high doses of chemical activators, for instance, could induce cell death possibly mediated by overstimulation of mitochondrial function.

The control of differentiation and of cell death could become interesting targets in the regulation of survival in neural precursors. Intense neuronal activity or neurodegenerative diseases increase oxidative environment finely modulated through TRP-induced homeostatic stability [76]. Then, the control of TRPV ion channels expression and activity on neural progenitors could trigger efficient signaling crosstalk mechanisms in response to oxidative stress, dysfunction, and damage of neural network [85, 86]. Specifically, the excitotoxicity might be modulated by calcium-dependent receptors internalization mediated by TRP ion channels. Thus, TRP expression and function might stimulate cell protection and regeneration on oxidative-stressed tissues [76, 87].

The transmission of electrical signals from neuron to neuron in complex networks and circuits is central to brain function. Cultured neural stem/progenitors may differentiate into neurons as a consequence of external neural activity [88]. This activity-dependent neurogenesis requires calcium channels in others, in proliferating stem/progenitor cells. Control of proliferation, survival, and connection on the brain network in neural precursors is key to obtain “excitation-neurogenesis coupling” [89], a perfect merge of the stem cell-derived newborn neurons with the remaining neurons in the brain. Thus, the controlled activation of TRPV1 might offer an innovative strategy to cover all these important aspects of well-functioning neural precursors.

## 7. Perspectives: Control of Neural Stem Cells through TRPV1

In the last years new techniques have been developed to monitor the activity of neural and nonneuronal cells remotely controlling ionic channels. The activation of specific cells through ionic channels triggers gene expression and peptide release* in vivo*. This constitutes a valuable research tool and a novel strategy for controlling cellular activity through regulated protein expression with potential applications in clinical settings.

The control of ionic channels depends on their biophysical properties as gating, desensitization, or allosteric coupling [90, 91]. These properties may be perturbed using chemicals ligands, voltage and light activation (optogenetics), physical modulation by temperature in specific points, or even magnetic fields [57, 92–94]. Some of these mechanisms are more convenient than others; chemical drug use is the simplest but the interaction with the entire organism in* in vivo* experiments leads to secondary effects or nonspecific results. On the other hand, the control of complex networks in animals through electrical or optical methods is technically challenging, because the depth of the tissues strongly attenuates electrical fields and light emission [92]. Magnetic fields were described as a specific remote control method because they have good penetration in biological tissues due to weak interaction with biological molecules [92, 94]. This weak interaction implies that in cells the magnetic fields have to be converted into a different stimulus such as aggregation of particles or mechanical force to act on their targets. One of the most common uses is the coupling of metal nanoparticles to ionic channels; these nanoparticles absorb energy and heat in response to radiofrequencies [94–96]. Using a temperature-sensitive channel, the heating can be converted into a cellular signal to allow ion influx and control of cellular functions [94].

TRPV1 have been used to control specific functions in neuronal and nonneuronal context in culture and in behaving animals [56, 57, 91, 97, 98]. In pancreas, a modified TRPV1 with antibody-coated iron oxide nanoparticles has been used as a temperature sensor that gates calcium to stimulate synthesis and release of bioengineered insulin via a promoter sensitive to calcium when heated in a low-frequency magnetic field [94]. This is particularly interesting because radio waves can be used to remotely activate insulin secretion by heating both externally applied and endogenously synthesized nanoparticles. Nowadays it is possible to generate cells with the ability to produce nanoparticles and of being controlled by TRPV1 activation, at least in nonneural context. However, the selective TRPV1 expression-mediated activation of neurons has shown to produce fast activity onset and consequent behavioral responses that depend on the specified neural population [57]. A paradigm of control where TRPV1-induced neural activity reached peaks within 7 minutes lasted only 10 minutes and was repeated immediately, stimulating brain plasticity through neurotransmitter release of dopaminergic or serotonergic neurons in freely moving mice [57].

Theoretically, selective control of stem cells could be exploited to investigate mechanistic pathways of excitation-neurogenesis coupling [99]. The development of functional TRPV1 channels in our stem cells-derived neurons or in neural precursors may provide a strategy to remotely control the survival, differentiation, and plasticity of these cells.

Imagine there is a new regeneration medicine of stem cells with genetically modified low radiofrequency-sensitive TRPV1 channels to control the survival and integration of stem cells grafts in the human brain.

## References

[1] Lieberman A., Krishnamurthi N. (2013). Is there room for non-dopaminergic treatment in Parkinson disease?. *Journal of Neural Transmission*.

[2] Braak H., Del Tredici K. (2008). Assessing fetal nerve cell grafts in Parkinson's disease. *Nature Medicine*.

[3] Emgård M., Karlsson J., Hansson O., Brundin P. (1999). Patterns of cell death and dopaminergic neuron survival in intrastriatal nigral grafts. *Experimental Neurology*.

[4] Brauchi S., Orta G., Salazar M., Rosenmann E., Latorre R. (2006). A hot-sensing cold receptor: C-terminal domain determines thermosensation in transient receptor potential channels. *The Journal of Neuroscience*.

[5] Cristino L., de Petrocellis L., Pryce G., Baker D., Guglielmotti V., Di Marzo V. (2006). Immunohistochemical localization of cannabinoid type 1 and vanilloid transient receptor potential vanilloid type 1 receptors in the mouse brain. *Neuroscience*.

[6] Medvedeva Y. V., Kim M.-S., Usachev Y. M. (2008). Mechanisms of prolonged presynaptic Ca^2+^ signaling and glutamate release induced by TRPV1 activation in rat sensory neurons. *The Journal of Neuroscience*.

[7] Miller B. A. (2006). The role of TRP channels in oxidative stress-induced cell death. *Journal of Membrane Biology*.

[8] Venkatachalam K., Montell C. (2007). TRP channels. *Annual Review of Biochemistry*.

[9] Voets T., Talavera K., Owsianik G., Nilius B. (2005). Sensing with TRP channels. *Nature Chemical Biology*.

[10] EL Andaloussi-Lilja J., Lundqvist J., Forsby A. (2009). TRPV1 expression and activity during retinoic acid-induced neuronal differentiation. *Neurochemistry International*.

[11] Guo S.-Y., Yang G.-P., Jiang D.-J. (2008). Protection of capsaicin against hypoxia-reoxygenation-induced apoptosis of rat hippocampal neurons. *Canadian Journal of Physiology and Pharmacology*.

[12] Musella A., De Chiara V., Rossi S. (2009). TRPV1 channels facilitate glutamate transmission in the striatum. *Molecular and Cellular Neuroscience*.

[13] Waning J., Vriens J., Owsianik G. (2007). A novel function of capsaicin-sensitive TRPV1 channels: involvement in cell migration. *Cell Calcium*.

[14] Goswami C., Schmidt H., Hucho F. (2007). TRPV1 at nerve endings regulates growth cone morphology and movement through cytoskeleton reorganization. *The FEBS Journal*.

[15] Goswami C., Hucho T. (2007). TRPV1 expression-dependent initiation and regulation of filopodia. *Journal of Neurochemistry*.

[16] Marinelli S., Pascucci T., Bernardi G., Puglisi-Allegra S., Mercuri N. B. (2005). Activation of TRPV1 in the VTA excites dopaminergic neurons and increases chemical- and noxious-induced dopamine release in the nucleus accumbens. *Neuropsychopharmacology*.

[17] Jin K., Xie L., Kim S. H. (2004). Defective adult neurogenesis in CB1 cannabinoid receptor knockout mice. *Molecular Pharmacology*.

[18] Stock K., Garthe A., de Almeida Sassi F., Glass R., Wolf S. A., Kettenmann H. (2014). The capsaicin receptor TRPV1 as a novel modulator of neural precursor cell proliferation. *Stem Cells*.

[19] Park E. S., Kim S. R., Jin B. K. (2012). Transient receptor potential vanilloid subtype 1 contributes to mesencephalic dopaminergic neuronal survival by inhibiting microglia-originated oxidative stress. *Brain Research Bulletin*.

[20] Pegorini S., Braida D., Verzoni C. (2005). Capsaicin exhibits neuroprotective effects in a model of transient global cerebral ischemia in Mongolian gerbils. *British Journal of Pharmacology*.

[21] Pegorini S., Zani A., Braida D., Guerini-Rocco C., Sala M. (2006). Vanilloid VR1 receptor is involved in rimonabant-induced neuroprotection. *British Journal of Pharmacology*.

[22] Clapham D. E., Runnels L. W., Strübing C. (2001). The TRP ion channel family. *Nature Reviews Neuroscience*.

[23] Nishihara E., Hiyama T. Y., Noda M. (2011). Osmosensitivity of transient receptor potential vanilloid 1 is synergistically enhanced by distinct activating stimuli such as temperature and protons. *PLoS ONE*.

[24] Simon F., Leiva-Salcedo E., Armisén R. (2010). Hydrogen peroxide removes TRPM4 current desensitization conferring increased vulnerability to necrotic cell death. *The Journal of Biological Chemistry*.

[25] Beech D. J. (2012). Integration of transient receptor potential canonical channels with lipids. *Acta Physiologica*.

[26] Yamamoto S., Takahashi N., Mori Y. (2010). Chemical physiology of oxidative stress-activated TRPM2 and TRPC5 channels. *Progress in Biophysics and Molecular Biology*.

[27] Caterina M. J., Schumacher M. A., Tominaga M., Rosen T. A., Levine J. D., Julius D. (1997). The capsaicin receptor: a heat-activated ion channel in the pain pathway. *Nature*.

[28] Latorre R., Brauchi S., Orta G., Zaelzer C., Vargas G. (2007). ThermoTRP channels as modular proteins with allosteric gating. *Cell Calcium*.

[29] Brauchi S., Orta G., Mascayano C. (2007). Dissection of the components for PIP_2_ activation and thermosensation in TRP channels. *Proceedings of the National Academy of Sciences of the United States of America*.

[30] Ross R. A. (2003). Anandamide and vanilloid TRPV1 receptors. *British Journal of Pharmacology*.

[31] Poblete H., Oyarzún I., Olivero P. (2015). Molecular determinants of phosphatidylinositol 4,5-bisphosphate (PI(4,5)P_2_) binding to transient receptor potential V1 (TRPV1) channels. *The Journal of Biological Chemistry*.

[32] Picazo-Juarez G., Romero-Suarez S., Nieto-Posadas A. (2011). Identification of a binding motif in the S5 helix that confers cholesterol sensitivity to the TRPV1 ion channel. *The Journal of Biological Chemistry*.

[33] Chen S.-C., Chang T.-J., Wu F.-S. (2004). Competitive inhibition of the capsaicin receptor-mediated current by dehydroepiandrosterone in rat dorsal root ganglion neurons. *Journal of Pharmacology and Experimental Therapeutics*.

[34] Lu Y.-C., Chen C.-W., Wang S.-Y., Wu F.-S. (2009). 17*β*-Estradiol mediates the sex difference in capsaicin-induced nociception in rats. *The Journal of Pharmacology and Experimental Therapeutics*.

[35] Bollimuntha S., Selvaraj S., Singh B. (2011). Emerging roles of canonical TRP channels in neuronal function. *Advances in Experimental Medicine and Biology*.

[36] Chávez A. E., Chiu C. Q., Castillo P. E. (2010). TRPV1 activation by endogenous anandamide triggers postsynaptic long-term depression in dentate gyrus. *Nature Neuroscience*.

[37] Nilius B., Voets T. (2005). TRP channels: a TR(I)P through a world of multifunctional cation channels. *Pflügers Archiv*.

[38] Bernabò N., Pistilli M. G., Mattioli M., Barboni B. (2010). Role of TRPV1 channels in boar spermatozoa acquisition of fertilizing ability. *Molecular and Cellular Endocrinology*.

[39] Tóth A., Boczán J., Kedei N. (2005). Expression and distribution of vanilloid receptor 1 (TRPV1) in the adult rat brain. *Molecular Brain Research*.

[40] Wang L., Wang D. H. (2005). TRPV1 gene knockout impairs postischemic recovery in isolated perfused heart in mice. *Circulation*.

[41] Alawi K., Keeble J. (2010). The paradoxical role of the transient receptor potential vanilloid 1 receptor in inflammation. *Pharmacology and Therapeutics*.

[42] Szallasi A., Nilsson S., Farkas-Szallasi T., Blumberg P. M., Hökfelt T., Lundberg J. M. (1995). Vanilloid (capsaicin) receptors in the rat: distribution in the brain, regional differences in the spinal cord, axonal transport to the periphery, and depletion by systemic vanilloid treatment. *Brain Research*.

[43] Cavanaugh D. J., Chesler A. T., Jackson A. C. (2011). *Trpv1* reporter mice reveal highly restricted brain distribution and functional expression in arteriolar smooth muscle cells. *Journal of Neuroscience*.

[44] Zschenderlein C., Gebhardt C., und Halbach O. V. B., Kulisch C., Albrecht D. (2011). Capsaicin-induced changes in LTP in the lateral amygdala are mediated by TRPV1. *PLoS ONE*.

[45] Martins D., Tavares I., Morgado C. (2014). ‘Hotheaded’: the role of TRPV1 in brain functions. *Neuropharmacology*.

[46] Mezey É., Tóth Z. E., Cortright D. N. (2000). Distribution of mRNA for vanilloid receptor subtype 1 (VR1), and VR1-like immunoreactivity, in the central nervous system of the rat and human. *Proceedings of the National Academy of Sciences of the United States of America*.

[47] Han P., Korepanova A. V., Vos M. H., Moreland R. B., Chiu M. L., Faltynek C. R. (2013). Quantification of TRPV1 protein levels in rat tissues to understand its physiological roles. *Journal of Molecular Neuroscience*.

[48] Sanchez J. F., Krause J. E., Cortright D. N. (2001). The distribution and regulation of vanilloid receptor VR1 and VR1 5′ splice variant RNA expression in rat. *Neuroscience*.

[49] Kauer J. A., Gibson H. E. (2009). Hot flash: TRPV channels in the brain. *Trends in Neurosciences*.

[50] Huang W., Yu F., Sanchez R. M. (2015). TRPV1 promotes repetitive febrile seizures by pro-inflammatory cytokines in immature brain. *Brain, Behavior, and Immunity*.

[51] Wianny F., Bernat A., Huissoud C. (2008). Derivation and cloning of a novel rhesus embryonic stem cell line stably expressing tau-green fluorescent protein. *STEM CELLS*.

[52] Wianny F., Bourillot P.-Y., Dehay C. (2011). Embryonic stem cells in non-human primates: an overview of neural differentiation potential. *Differentiation*.

[53] Biggs J. E., Yates J. M., Loescher A. R., Clayton N. M., Boissonade F. M., Robinson P. P. (2007). Changes in vanilloid receptor 1 (TRPV1) expression following lingual nerve injury. *European Journal of Pain*.

[54] Goswami C., Hucho T. (2008). Submembraneous microtubule cytoskeleton: biochemical and functional interplay of TRP channels with the cytoskeleton. *FEBS Journal*.

[55] Nagy I., Sántha P., Jancsó G., Urbán L. (2004). The role of the vanilloid (capsaicin) receptor (TRPV1) in physiology and pathology. *European Journal of Pharmacology*.

[56] Arenkiel B. R., Klein M. E., Davison I. G., Katz L. C., Ehlers M. D. (2008). Genetic control of neuronal activity in mice conditionally expressing TRPV1. *Nature Methods*.

[57] Güler A. D., Rainwater A., Parker J. G. (2012). Transient activation of specific neurons in mice by selective expression of the capsaicin receptor. *Nature Communications*.

[58] Mori F., Ribolsi M., Kusayanagi H. (2012). TRPV1 channels regulate cortical excitability in humans. *The Journal of Neuroscience*.

[59] Gibson H. E., Edwards J. G., Page R. S., Van Hook M. J., Kauer J. A. (2008). TRPV1 channels mediate long-term depression at synapses on hippocampal interneurons. *Neuron*.

[60] Shoudai K., Peters J. H., McDougall S. J., Fawley J. A., Andresen M. C. (2010). Thermally active TRPV1 tonically drives central spontaneous glutamate release. *The Journal of Neuroscience*.

[61] Marsch R., Foeller E., Rammes G. (2007). Reduced anxiety, conditioned fear, and hippocampal long-term potentiation in transient receptor potential vanilloid type 1 receptor-deficient mice. *The Journal of Neuroscience*.

[62] Chávez A. E., Hernández V. M., Rodenas-Ruano A., Savio Chan C., Castillo P. E. (2014). Compartment-specific modulation of GABAergic synaptic transmission by TRPV1 channels in the dentate gyrus. *Journal of Neuroscience*.

[63] Marinelli S., Di Marzo V., Berretta N. (2003). Presynaptic facilitation of glutamatergic synapses to dopaminergic neurons of the rat substantia nigra by endogenous stimulation of vanilloid receptors. *Journal of Neuroscience*.

[64] Marinelli S., Di Marzo V., Florenzano F. (2007). N-arachidonoyl-dopamine tunes synaptic transmission onto dopaminergic neurons by activating both cannabinoid and vanilloid receptors. *Neuropsychopharmacology*.

[65] Komuro H., Rakic P. (1996). Intracellular Ca^2+^ fluctuations modulate the rate of neuronal migration. *Neuron*.

[66] Gu X., Olson E. C., Spitzer N. C. (1994). Spontaneous neuronal calcium spikes and waves during early differentiation. *The Journal of Neuroscience*.

[67] Weissman T. A., Riquelme P. A., Ivic L., Flint A. C., Kriegstein A. R. (2004). Calcium waves propagate through radial glial cells and modulate proliferation in the developing neocortex. *Neuron*.

[68] Fiorio Pla A., Maric D., Brazer S.-C. (2005). Canonical transient receptor potential 1 plays a role in basic fibroblast growth factor (bFGF)/FGF receptor-1-induced Ca^2+^ entry and embryonic rat neural stem cell proliferation. *The Journal of Neuroscience*.

[69] Zheng J. Q., Poo M.-M. (2007). Calcium signaling in neuronal motility. *Annual Review of Cell and Developmental Biology*.

[70] Goswami C., Dreger M., Otto H., Schwappach B., Hucho F. (2006). Rapid disassembly of dynamic microtubules upon activation of the capsaicin receptor TRPV1. *Journal of Neurochemistry*.

[71] Hu F., Sun W. W., Zhao X. T., Cui Z. J., Yang W. X. (2008). TRPV1 mediates cell death in rat synovial fibroblasts through calcium entry-dependent ROS production and mitochondrial depolarization. *Biochemical and Biophysical Research Communications*.

[72] Shirakawa H., Yamaoka T., Sanpei K., Sasaoka H., Nakagawa T., Kaneko S. (2008). TRPV1 stimulation triggers apoptotic cell death of rat cortical neurons. *Biochemical and Biophysical Research Communications*.

[73] Macho A., Blazquez M., Navas P. (1998). Induction of apoptosis by vanilloid compounds does not require de novo gene transcription and activator protein 1 activity. *Cell Growth & Differentiation*.

[74] Macho A., Lucena C., Calzado M. (2000). Phorboid 20-homovanillates induce apoptosis through a VR1-independent mechanism. *Chemistry & Biology*.

[75] Mizrak S. C., van Dissel-Emiliani F. M. F. (2008). Transient receptor potential vanilloid receptor-1 confers heat resistance to male germ cells. *Fertility and Sterility*.

[76] Wang M., Ji P., Wang R., Zhao L., Xia Z. (2012). TRPV1 agonist capsaicin attenuates lung ischemia-reperfusion injury in rabbits. *Journal of Surgical Research*.

[77] Touska F., Marsakova L., Teisinger J., Vlachova V. (2011). A ‘cute’ desensitization of TRPV1. *Current Pharmaceutical Biotechnology*.

[78] Sanz-Salvador L., Andrés-Borderia A., Ferrer-Montiel A., Planells-Cases R. (2012). Agonist- and Ca^2+^-dependent desensitization of TRPV1 channel targets the receptor to lysosomes for degradation. *The Journal of Biological Chemistry*.

[79] Storti B., Di Rienzo C., Cardarelli F., Bizzarri R., Beltram F., Phillips W. (2015). Unveiling TRPV1 spatio-temporal organization in live cell membranes. *PLoS ONE*.

[80] Dedov V. N., Mandadi S., Armati P. J., Verkhratsky A. (2001). Capsaicin-induced depolarisation of mitochondria in dorsal root ganglion neurons is enhanced by vanilloid receptors. *Neuroscience*.

[81] Wood J. N., Winter J., James I. F., Rang H. P., Yeats J., Bevan S. (1988). Capsaicin-induced ion fluxes in dorsal root ganglion cells in culture. *Journal of Neuroscience*.

[82] Lang H., Li Q., Yu H. (2015). Activation of TRPV1 attenuates high salt-induced cardiac hypertrophy through improvement of mitochondrial function. *British Journal of Pharmacology*.

[83] Tao R., Vassilopoulos A., Parisiadou L., Yan Y., Gius D. (2014). Regulation of MnSOD enzymatic activity by Sirt3 connects the mitochondrial acetylome signaling networks to aging and carcinogenesis. *Antioxidants and Redox Signaling*.

[84] Hao X., Chen J., Luo Z. (2011). TRPV1 activation prevents high-salt diet-induced nocturnal hypertension in mice. *Pflügers Archiv*.

[85] Maione S., Cristino L., Migliozzi A. L. (2009). TRPV1 channels control synaptic plasticity in the developing superior colliculus. *Journal of Physiology*.

[86] Ryu V., Gallaher Z., Czaja K. (2010). Plasticity of nodose ganglion neurons after capsaicin- and vagotomy-induced nerve damage in adult rats. *Neuroscience*.

[87] Marchalant Y., Brothers H. M., Norman G. J., Karelina K., DeVries A. C., Wenk G. L. (2009). Cannabinoids attenuate the effects of aging upon neuroinflammation and neurogenesis. *Neurobiology of Disease*.

[88] Deisseroth K., Singla S., Toda H., Monje M., Palmer T. D., Malenka R. C. (2004). Excitation-neurogenesis coupling in adult neural stem/progenitor cells. *Neuron*.

[89] Hsieh J., Schneider J. W. (2013). Neural stem cells, excited. *Science*.

[90] Jara-Oseguera A., Islas L. D. (2013). The role of allosteric coupling on thermal activation of thermo-TRP channels. *Biophysical Journal*.

[91] Zemelman B. V., Nesnas N., Lee G. A., Miesenböck G. (2003). Photochemical gating of heterologous ion channels: remote control over genetically designated populations of neurons. *Proceedings of the National Academy of Sciences of the United States of America*.

[92] Huang H., Delikanli S., Zeng H., Ferkey D. M., Pralle A. (2010). Remote control of ion channels and neurons through magnetic-field heating of nanoparticles. *Nature Nanotechnology*.

[93] Johansen J. P., Hamanaka H., Monfils M. H. (2010). Optical activation of lateral amygdala pyramidal cells instructs associative fear learning. *Proceedings of the National Academy of Sciences of the United States of America*.

[94] Stanley S. A., Gagner J. E., Damanpour S., Yoshida M., Dordick J. S., Friedman J. M. (2012). Radio-wave heating of iron oxide nanoparticles can regulate plasma glucose in mice. *Science*.

[95] Hamad-Schifferli K., Schwartz J. J., Santos A. T., Zhang S., Jacobson J. M. (2002). Remote electronic control of DNA hybridization through inductive coupling to an attached metal nanocrystal antenna. *Nature*.

[96] Richardson H. H., Hickman Z. N., Thomas A. C., Kordesch M. E., Govorov A. O. (2006). *Thermo-Optical Properties of Nanoparticles and Nanoparticle Complexes Embedded in Ice: Characterization of Heat Generation and Actuation of Larger-Scale Effects*.

[97] Donlea J. M., Thimgan M. S., Suzuki Y., Gottschalk L., Shaw P. J. (2011). Inducing sleep by remote control facilitates memory consolidation in *Drosophila*. *Science*.

[98] Tobin D. M., Madsen D. M., Kahn-Kirby A. (2002). Combinatorial expression of TRPV channel proteins defines their sensory functions and subcellular localization in *C. elegans* neurons. *Neuron*.

[99] Hsieh J., Schneider J. W. (2013). Neural stem cells, excited. *Science*.

